# Pregnancy-induced differential expression of SARS-CoV-2 and influenza a viral entry factors in the lower respiratory tract

**DOI:** 10.1371/journal.pone.0281033

**Published:** 2023-07-12

**Authors:** Tusar Giri, Santosh Panda, Arvind Palanisamy

**Affiliations:** 1 Department of Anesthesiology, Washington University School of Medicine, St. Louis, MO, United States of America; 2 Department of Pathology, Washington University School of Medicine, St. Louis, MO, United States of America; 3 Department of Obstetrics and Gynecology, Washington University School of Medicine, St. Louis, MO, United States of America; The University of Texas Medical Branch at Galveston, UNITED STATES

## Abstract

Despite differences in the clinical presentation of coronavirus disease-19 and pandemic influenza in pregnancy, fundamental mechanistic insights are currently lacking because of the difficulty in recruiting critically ill pregnant subjects for research studies. Therefore, to better understand host-pathogen interaction during pregnancy, we performed a series of foundational experiments in pregnant rats at term gestation to assess the expression of host entry factors for severe acute respiratory syndrome coronavirus 2 (SARS-CoV-2) and influenza A virus (IAV) and genes associated with innate immune response in the lower respiratory tract. We report that pregnancy is characterized by a decrease in host factors mediating SARS-CoV-2 entry and an increase in host factors mediating IAV entry. Furthermore, using flow cytometric assessment of immune cell populations and immune provocation studies, we show an increased prevalence of plasmacytoid dendritic cells and a Type I interferon-biased environment in the lower respiratory tract of pregnancy, contrary to the expected immunological indolence. Our findings, therefore, suggest that the dissimilar clinical presentation of COVID-19 and pandemic influenza A in pregnancy could partly be due to differences in the extent of innate immune activation from altered viral tropism and indicate the need for comparative mechanistic investigations with live virus studies.

## Introduction

The varied clinical presentation of respiratory pandemics in pregnancy is an unresolved puzzle. Compared to the higher mortality rates reported with pandemic influenza A in pregnancy [1,2], the high rate of asymptomatic infection and the 0–1% mortality rate of the current coronavirus disease-19 (COVID-19) pandemic in pregnancy is striking [3–11]. Though pregnant women with symptomatic COVID-19 have a higher rate of admission to the ICU, even in this subset of critically ill women, the mortality rate was still approximately 1% [12]. Clinical studies in pregnant women focus on mortality, ICU morbidity, obstetric/neonatal outcomes, and placental transfer of the virus, but are unable to shed light on the mechanisms that can lead to these disparate clinical outcomes. Animal studies, in contrast, provide extensive mechanistic insights into the effects of respiratory viral infection on lung immunopathology and its resolution during pregnancy [13–16], but have not directly compared the outcomes of influenza A with COVID-19 infection.

A fundamental question that remains unaddressed is whether the state of pregnancy alters the expression of host cell entry factors for these causative viruses. This is an important question because the sex hormones estradiol and progesterone are known to influence the expression of SARS-CoV-2 and influenza A entry factors [17–20]. Whether the sustained multi-fold increase in estradiol and progesterone during pregnancy produces a similar change in the expression of viral entry factors remains unstudied. Building upon our previous work using the pregnant rat as a model organism [21,22], we evaluated the lung, the ultimate effector organ for pathology and disease severity, for pregnancy-induced changes in the expression of entry factors for the viruses of interest (influenza A virus [IAV] and severe acute respiratory syndrome coronavirus 2 [SARS-CoV-2], respectively). We further assessed the expression of genes associated with innate immune defense, relative frequencies of immune cells, and the magnitude of type 1 interferon (IFN-1) response. Here, we provide novel evidence for the differential expression of host cell entry factors for influenza A and SARS-CoV-2 during pregnancy, with upregulated innate immune defense in the lower respiratory tract. Our findings raise the possibility of differential susceptibility to infection during pregnancy and provide the scientific rationale for pursuing these studies in pregnant women.

## Materials and methods

All experiments were conducted after appropriate institutional approval (protocol ID:19–1071; Institutional Animal Care and Use Committee, Washington University School of Medicine, St. Louis, MO) and were performed in full accordance with both institutional regulations and the ARRIVE (Animals in Research: Reporting *In Vivo* Experiments) guidelines. Because of the pregnancy-specific nature of the research question, only pregnant (gestational day GD20) and non-pregnant adult female rats were used in the study (CD^®^ Sprague Dawley IGS strain, Charles River Laboratories). Group sizes (n = 6–9) were determined based on the effect sizes observed during our previous studies [21,22]. For molecular biology and pregnancy-related tissue hormone assays, lungs were collected from GD20 pregnant and non-pregnant female rats after bilateral thoracotomy under deep isoflurane anesthesia followed by perfusion through the right ventricle with up to 30 mL of ice cold 1x PBS, and stored at -80°C. To ensure consistency, these experiments were performed with tissue from the lower lobe of the right lung.

### Comparative assessment of viral entry factors in pregnancy

Pregnancy is characterized by an 3–5 fold increase in plasma estradiol and progesterone [23], both of which have considerable influence on the expression of viral entry receptors and associated immune response [16,17,19,24–28]. Because the lung tissue concentration of these hormones in pregnancy is unknown, we assayed for 17β-estradiol (LS-F55510, LifeSpan BioSciences, Inc.) and progesterone (LS-F39173, LifeSpan BioSciences, Inc.) as described by us previously [21]. Next, we performed TaqMan gene expression studies to assess the expression of the canonical host cell entry receptors for SARS-CoV-2 (angiotensin converting enzyme-2 [ACE2] and transmembrane serine protease 2 [TMPRSS2]) and IAV (sialic acid [SA] α-2,3- and α-2,6-linked glycans for avian and human influenza, respectively) as described previously [21,22]. Simultaneously, we performed immunoblotting experiments for these receptors as described previously [21,22]. Briefly, we determined the expression of ACE2 (LS-c763699, LifeSpan BioSciences, Inc.; 1:1000 dilution) and TMPRSS2 (sc-515727, Santa Cruz Biotechnology, Inc.; 1:250 dilution) protein with immunoblots. Human ACE2 and TMPRSS2 expressed in HEK293 cells served as positive controls and rat intestinal lysate was included as an additional control. Next, we assayed ACE2 enzyme activity with a fluorometric assay (K897-100, Biovision Inc.) according to manufacturer’s instructions [22]. For IAV receptor expression, we used the anti-ST3GAL4 antibody (#MBS9133580, MyBioSource Inc.; 1:250 dilution) and anti-ST6GAL1 antibody (#MBS3216285, MyBioSource Inc.; 1:500 dilution) for α-2,3- and α-2,6-linked glycans, respectively. We confirmed this with highly specific lectin-based flow cytometry experiments to directly evaluate the presence of IAV receptors on EpCAM+ve epithelial cells as described by us previously [21], using the following antibodies: FITC*SNA-I (Sambucus nigra agglutinin-I; #F-6802-1, EY Laboratories Inc.), Biotin*MAA (Maackia amurensis agglutinin; #BA-7801-2, EY Laboratories Inc.), Brilliant Violet 421*Streptavidin (#405226, BioLegend, Inc.), mAb-EpCAM (GZ-1) (#ab187276, Abcam plc.), and APC Goat anti-mouse IgG (#405308, BioLegend, Inc.).

### Expression of innate antiviral defense genes in pregnancy

To obtain a nuanced perspective of pregnancy-induced changes in innate immune genes, we sampled multiple unrelated and overlapping canonical immune pathways to encompass both virus-specific and non-specific genes involved in antiviral defense. First, we assayed the expression levels of genes related to the TLR (toll-like receptor)-7 (*Tlr7*, *MyD88*, *Irf7*) and TLR-3 pathways (*Tlr3*, *Traf3*, *Irf3*) necessary for the immune response against SARS-CoV-2 and IAV, respectively [29]. Next, we assayed the expression of the most common innate immune genes that are co-expressed in epithelial cells expressing the ACE2 receptor (*Irak3*, *Ido1*, *Tnfsf10*, *Mx1*, *Nos2*) [30]. Finally, we assayed the cytosolic immune sensor *Rig-1* and emblematic inflammation-associated genes (*Nfkb1*, *Nfkb2*, and *IL6*). All Taqman assays were performed in duplicate along with two endogenous housekeeping control genes (*Eef2* and *Actb*) as described previously [21,22].

### Flow cytometric identification of innate immune cell types

Single cell suspensions were prepared from isolated lungs and stained for flow cytometry as described for nasal epithelial tissue [21]. Following fluorescent-labeled antibodies were used: APC/cy7*anti-rat CD45 (#202216, BD Biosciences), Brilliant Violet 421*anti-rat CD3 clone 1F4 (RV0) (#563948, BD Biosciences), APC*anti-rat CD11b clone WTS (RV0) (#562102 BD Biosciences), FITC* anti-rat RT1Dab (MHCII) (#205405, BioLegend, Inc.), PerCP/cy 5.5* anti-rat CD4 (#201519, BioLegend, Inc.), and PE* anti-rat CD161 (#205604, BioLegend Inc.). Live lymphocyte sized cells were stained with CD45 and CD3 to identify T cells. Further, the T cells were classified into T helper and cytotoxic T cells based on CD4 and CD8 expression, respectively. CD3-, CD11b-, CD4+ and MHCII+ cells were identified as plasmacytoid dendritic cells (pDCs). CD3- and CD161+ cells were identified as natural killer (NK) cells. Gating strategies have been elaborated in detail in our previous publication [21].

### Immune provocation studies with intranasal resiquimod

Based on our previous observations of enhanced Type 1 interferon (IFN-1) response in the nasal epithelium after chemical provocation with intranasal resiquimod (R848) [21], a synthetic TLR-7/8 agonist, we sought to examine whether this was true for the pregnant lung. Mildly anesthetized (isoflurane 2% for 3–4 min) dams and non-pregnant rats were treated with intranasal resiquimod as described by us previously [21]. The choice of 2-hour timepoint was guided by previous work in mice and humans showing maximal Type I interferon response at 2–3 hours after R-848 [31–33]. After 2 h, the animals were subjected to bronchoalveolar lavage (BAL) followed by lung collection under pentobarbital euthanasia (100 mg/kg i.p.) for interferon and cytokine assays. BAL was performed as described by Wang et al. with modifications (using 2 mL of ice cold sterile PBS) [34]. We retrieved approximately 1.2 mL of BAL fluid on average for the cytokine assays. The concentrations of IFN-α and IFN-β in lung homogenates were measured using the Rat Interferon Alpha ELISA kit (#MBS4500053, MyBioSource Inc.) and Rat Interferon Beta ELISA kit (#MBS4500062, MyBioSource, Inc.), respectively, as described by us previously [21]. Quantitative measurement of 10 cytokines (IFN-γ, IL-1a, IL-1b, IL-2, IL-4, IL-6, IL-10, IL-13, MCP-1, and TNFα) was performed in BAL fluid and lung homogenates using the Quantibody^®^ Rat Inflammation Array Q1 kit (# QAR-INF-1, RayBiotech Life Inc.), according to manufacturers’ instructions [21]. Data extraction (GAL file) was done using microarray analysis software (GenePix^®^ Pro Microarray Analysis Software, Molecular Devices LLC).

### Statistical analysis

Normally and non-normally distributed data (except array data) were analyzed with Welch’s t-test and Mann-Whitney U test, respectively. Interferon release data were analyzed with 2-way ANOVA. Data other than cytokine array data were analyzed with Prism 9 for macOS (version 9.1.2; GraphPad Software Inc.) and presented as mean ± SD; p ≤ 0.05 was accorded statistical significance. Analyses of the Quantibody cytokine array data were performed in the array-specific Q-Analyzer Tool (QAR-INF-1-SW, RayBiotech Life Inc.) as described by us recently [21], and presented as mean ± SD or as median with minimum and maximum values.

## Results

### Differential expression of SARS-CoV-2 and IAV host entry factors in the pregnant lung

**Fig 1:**
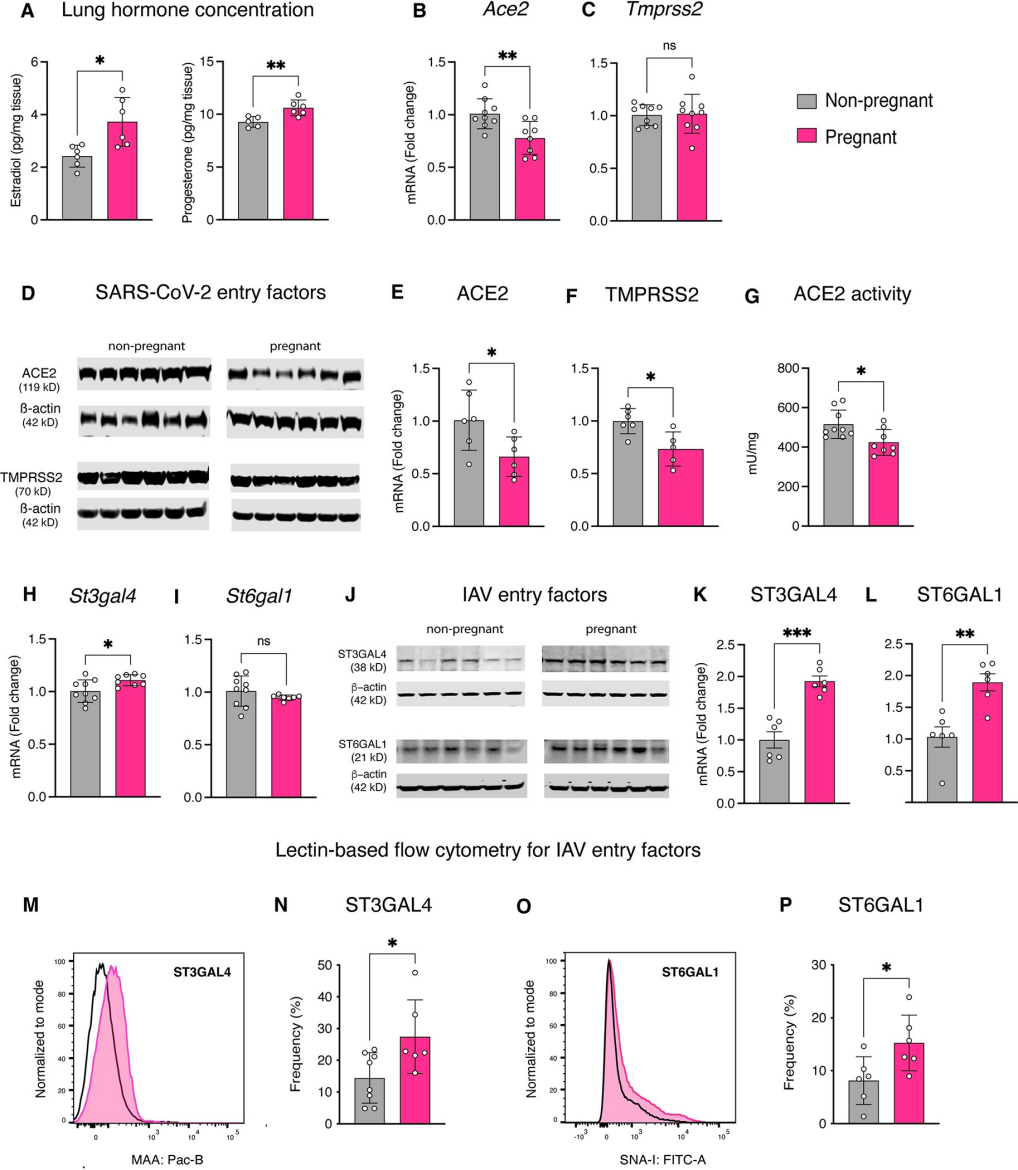
Differential expression of SARS-CoV-2 and IAV host entry factors in the pregnant lung. Scatter plots showing increased concentration of estradiol and progesterone (A) and enhanced *Ace2* gene expression in the lungs of pregnant rats (B). *Tmprss2* gene expression was unchanged (C). Immunoblots (D) and accompanying densitometric quantitation showing reduced expression of ACE2 (E) and TMPRSS2 (F) protein in the lungs of pregnant rats. Scatter plots showing reduced ACE2 activity in the lungs of pregnant rats (G). In contrast, *St3gal4* gene expression was enhanced (H), but not that of St6gal1 (I). Immunoblots (J) and accompanying densitometric quantitation showing reduced expression of ST3GAL4 (K) and ST6GAL1 (L) protein in the lungs of pregnant rats. Representative flow cytometry histograms depicting the increased expression of MAA (ST3GAL4) and SNA-I (ST6GAL1) on the surface of lung epithelial cells of pregnant (shaded) *vs*. age matched non-pregnant rats (black-line), along with their respective frequency scatter plots (M-P). Blot images are cropped and rearranged with white space (non-pregnant vs. pregnant samples) for clarity. Uncropped full-length blots are available in the attached S2-S5 Figs of S1 File. Data were analyzed with Welch’s t-test and presented as mean ± SD (n = 6–9 per condition); *p ≤ 0.05, **p ≤ 0.01, ***p ≤ 0.001.

Both estradiol and progesterone were significantly elevated in the lung of pregnant *vs*. non-pregnant rats (Fig 1A). When the expression of genes for SARS-CoV-2 entry factors was assayed, *Ace2* was downregulated in pregnancy (Fig 1B) but not *Tmprss2* (Fig 1C). Western blotting, however, revealed a decrease in the expression of both ACE2 and TMPRSS2 protein in the lung of pregnant rats (Fig 1D–1F). These observations were accompanied by a decrease in ACE2 enzyme activity (Fig 1G). When IAV related entry factors were examined, we noted increased expression of the sialyltransferase enzyme for the avian IAV receptor, *St3gal4*, with pregnancy (Fig 1H). However, expression of the sialyltransferase enzyme for the human IAV receptor, *St6gal1*, was unchanged (Fig 1I). Western blotting, however, revealed increased expression of both ST3GAL4 and ST6GAL1 (Fig 1J–1L) which was confirmed with lectin-based flow cytometry showing a notable increase in both α-2,3- (Fig 1M–1N) and α-2,6-linked glycans (Fig 1O–1P), respectively. Uncropped western blots for the viral receptors of interest are provided in the S1–S4 Figs of S1 File.

### Enhanced antiviral state and upregulated pathogen sensing in the pregnant lung

**Fig 2:**
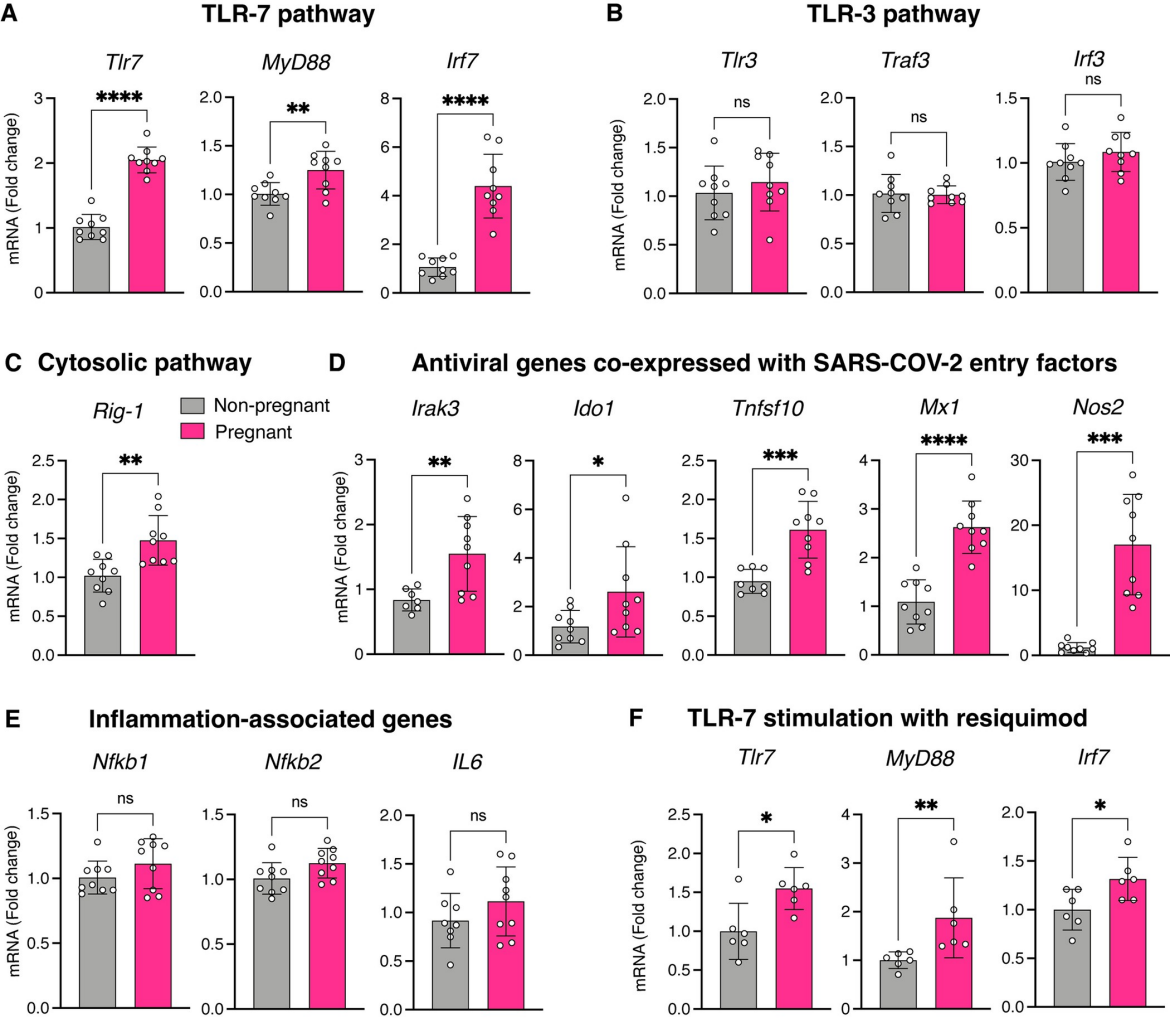
Enhanced antiviral state and upregulated pathogen sensing in the pregnant lung. Scatter plots showing enhanced expression of genes representing the TLR-7 (A) but not the TLR-3 pathway (B) in pregnancy. In addition, pregnancy was characterized by increased expression of the cytosolic immune sensor *Rig-1* (C) and common antiviral genes known to be co-expressed with SARS-CoV-2 entry factors (D), but not those associated with inflammation (E). Resiquimod administration significantly increased the expression of TLR-7 pathway genes in pregnancy (F). Data were analyzed with Welch’s t-test and presented as mean ± SD (n = 6–9 per condition); *p ≤ 0.05, **p ≤ 0.01, ***p ≤ 0.001, ****p ≤ 0.0001.

Because the innate immune system is the first line of defense against invading respiratory pathogens, we assessed the baseline state of innate immune defense in pregnancy. Genes related to the TLR-7 pathway (*Tlr7*, *MyD88*, *Irf7*) were upregulated (Fig 2A) but genes from the TLR-3 pathway (*Tlr3*, *Traf3*, *Irf3*) was largely unchanged (Fig 2B). The expression of cytosolic immune sensor *Rig-1* was markedly enhanced in pregnancy (Fig 2C). Non-specific antiviral genes that are highly co-expressed with SARS-CoV-2 entry factors (*Irak3*, *Ido1*, *Tnfsf10*, *Mx1*, *Nos2*) [30] were substantially upregulated in the pregnant lung (Fig 2D). However, the expression of inflammation-associated genes (*Nfkb1*, *Nfkb2*, and *IL6*) were comparable between pregnant *vs*. non-pregnant lungs (Fig 2E). When stimulated with the TLR-7 agonist resiquimod, emblematic TLR-7 pathway genes were markedly upregulated in pregnant *vs*. non-pregnant rats (Fig 2F).

### Immune cell census and response to immune stimulation are altered in the pregnant lung

**Fig 3:**
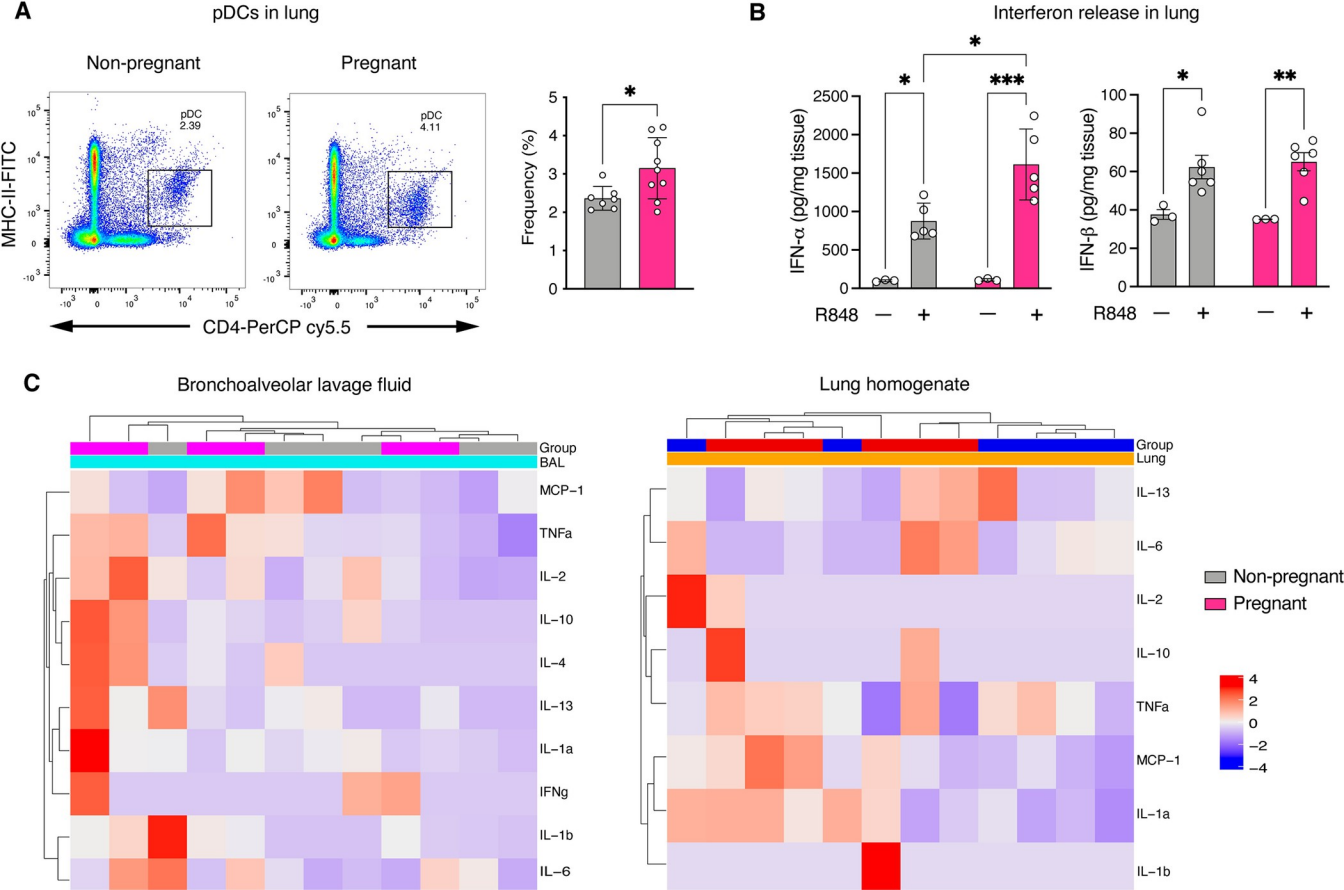
Immune cell census and response to immune stimulation are altered in the pregnant lung. Representative FACS two-parameter pseudocolor dot plot and frequency scatter plot to the right (A) depicting increased presence of pDCs (area marked with square representing the percentages of pDCs) in pregnancy. Gating for pDC was performed as outlined in our previous work [21]. Though resiquimod (R848) increased both IFN-α and IFN-β in both groups, the increase in IFN-α was more marked in pregnant rats (B). For the cytokine experiments (C), the scaled and centered data were plotted as a heatmap in which the different colors represent cytokine expression levels. There were no significant differences in cytokine expression levels between the two groups in both BAL fluid and lung homogenates. Data were analyzed with either Welch’s t-test, 2-way ANOVA or Mann-Whitney U test and presented as mean ± SD (n = 6–9 each); *p ≤ 0.05, **p ≤ 0.01, ***p ≤ 0.001.

Flow cytometric assessment of immune cell frequencies in single cell suspensions of the lung revealed an increase in pDCs in the pregnant lung (Fig 3A); frequencies of CD4+, CD8+, and NK cells were unchanged (S5 Fig of S1 File). Immune stimulation with resiquimod was associated with enhanced release of IFN-α and IFN-β in the lungs of both pregnant and non-pregnant rats (Fig 3B). However, the magnitude of the increase in IFN-α was significantly higher in pregnant rats, while the IFN-β response was similar in pregnant *vs*. non-pregnant rats. We did not observe significant differences in the inflammatory cytokine response either in the BAL fluid or lung homogenates of both groups (Fig 3C and Table 1).

**Table 1:** Cytokine response in the lower respiratory tract 2 h after intranasal resiquimod.

	BAL		p value	Lung		p value
	Non-pregnant (pg/mL)	Pregnant (pg/mL)		Non-pregnant (pg/mL)	Pregnant (pg/ mL)	
IL-6	45 ± 39	53 ± 32	0.93	59.8 ± 43.3	72.4 ± 78.3	1.0
IL-4	0.4 (0.4, 0.9)	0.5 (0.4, 1.6)	0.77	0.4 ± 0	0.4 ± 0	1.0
IL-10	55 (55, 166)	91 (55, 371)	0.40	55 ± 0	67 ± 20	1.0
MCP-1	1551 ± 1521	1657 ± 1209	0.99	3992 ± 1120	6480 ± 2434	0.57
IL-13	11 (5.6, 42)	14 (5.6, 53)	0.93	19 ± 8	18 ± 7	1.0
TNF-α	18299 ± 7427	31871 ± 12277	0.40	15514 ± 2748	15308 ± 6400	1.0
IL-2	243 ± 140	382 ± 222	0.75	20 (20, 1002)	20 (20, 296)	1.0
IFN-γ	5.3 (5.3, 7.8)	5.3 (5.3, 9.8)	0.77	5.3 ± 0	5.3 ± 0	1.0
IL-1α	503 ± 338	1077 ± 1550	0.77	1379 ± 864	1714 ± 700	1.0
IL-1β	180 ± 381	181 ± 81	0.99	20 ± 0	20 ± 1	1.0

Data expressed as either mean ± S.D or as median with minimum and maximum values.

BAL: Bronchoalveolar lavage.

P values are false discovery rate (FDR)-adjusted.

## Discussion

In this report, we present the earliest comparative evidence for pregnancy-induced differential expression of viral entry factors for SARS-CoV-2 and IAV in the lower respiratory tract. Specifically, host factors mediating SARS-CoV-2 entry were downregulated while those for human and avian IAV were upregulated, raising the possibility of differential tissue tropism and potentially altered cellular viral load. Furthermore, we present evidence to confirm that pregnancy is characterized by an increase in innate immune gene expression preferentially involving the TLR-7 pathway and enhanced IFN-1 response in the lung. Because a preactivated innate immune state is protective against viral infections and an overactivated state leads to immunopathology [29,35], differences in cellular viral load could be an important driver of the observed variant mortality outcomes between COVID-19 and pandemic influenza A.

To our knowledge, our work is the first to show *in vivo* that pregnancy induces differential expression of the cell entry factors for SARS-CoV-2 and IAV in the lower respiratory tract. Foundational studies such as these are difficult in pregnant women because the lower respiratory tract is rarely accessible for research unless they are critically ill. Our observation of reduced ACE2 and TMPRSS2 expression in the lower respiratory tract during pregnancy is consistent with *in vitro* observations showing estradiol-mediated downregulation of these receptors [17,24,28], and with our previous work with nasal epithelial tissue [22]. Though hormonal changes of pregnancy are known to increase systemic sialylation [18,36–38], our study is the first to show upregulated sialic acid glycoproteins in the lower respiratory tract. These include sialic acid [SA] α-2,3, and α-2,6-linked glycans which show highly specific binding with either avian or human IAV [39–41], respectively. Contrasting this with decreased expression of SARS-CoV-2 entry factors, it is likely that viral attachment and cellular entry is potentially higher for IAV compared to SARS-CoV-2, a prediction that is best independently confirmed with *in vivo* live virus studies. The scientific rationale for pursuing these studies is solid with recent studies highlighting the correlation between high viral load and clinical disease severity for both SARS-CoV-2 and influenza A infections [42–48].

Our gene expression data provide several novel insights into mechanisms of disease resistance and pathogenesis in the pregnant lung. First, innate immune genes known to be co-expressed with ACE2/TMPRSS2 (*Ido1*, *Irak*, *Tnsfs1*, *NOS2*, *Mx1*) [30] were substantively upregulated, suggesting the possibility that pregnancy is characterized by an enhanced anti-SARS-CoV-2 state at baseline. Second, from a viral pathogen recognition standpoint, we noted a unique upregulation of genes selective to the TLR-7 (*Tlr7*, *MyD88*, *Irf7*), but not the TLR-3 pathway. Because of the critical importance of TLR-7 pathway in combating SARS-CoV-2 infection [49–51], we speculate that the enhanced immune sensing pathways confer a preactivated innate immune state ready to encounter SARS-CoV-2, similar to data recently reported in children [35]. Third, considering that the respiratory tract is vulnerable to exaggerated immunopathology during pregnancy [14], we were particularly intrigued by the marked overexpression of cytokine-inducible nitric oxide synthase (NOS2), an antiviral gene which, when profoundly activated, can cause severe oxidative lung injury. This is aptly illustrated in NOS2-deficient mice, where infection with IAV was associated with reduced mortality, morbidity, and decreased cytokine production in the lung [52,53]. Therefore, it is plausible that enhanced IAV load from increased cellular entry overwhelms the antiviral defense and activates NOS2 to cause increased oxidative injury and severe lung immunopathology during IAV infection in pregnancy. Collectively, our gene expression data suggest that the pregnant lung is potentially endowed with the ability to resist SARS-CoV-2 but worsen IAV infection and presents a nuanced picture that contrasts with the conventional paradigm of generalized immune suppression in pregnancy.

Qualitative assessment of innate immune cells confirmed an increase in pDCs in the lungs of pregnant rats, similar to our previous observations in the nasal epithelium [21]. Taken together with an enhanced IFN-1 response after immune stimulation with resiquimod, we speculate that the lower respiratory tract in pregnancy is characterized by a preactivated innate immune system that is primed to respond to invading pathogens. We envision a scenario where this preactivated innate immune system is highly effective when the viral load is low, as would occur from reduced cellular entry of SARS-CoV-2 but could lead to overactivation and immunopathology when the viral load is high, for example, from increased cellular entry of IAV. The absence of differences in inflammation-associated genes (*Nfkb1*, *Nfkb2*, *IL-6*) and the cytokine response after resiquimod suggests that pregnancy is not a pro-inflammatory state at baseline.

A major strength of our work is the simultaneous assessment of host factors in pregnancy for the two most pertinent respiratory viruses, namely, SARS-CoV-2 and IAV. Enhanced pathogen sensing ability through TLR-7 and a potentially preactivated innate immune state are novel discoveries that challenge the dogma of pregnancy-induced immunosuppression. Nevertheless, our study has a few important limitations. A major limitation is that we did not perform live virus studies because of the lack of a BSL-3 facility. Therefore, the possibility of differences in cellular viral entry remains to be tested. However, we anticipate that our work will inspire other investigators with access to these tools to perform comparative *in vivo* infection studies. Second, the convenient use of a pregnant rat model allows us to examine the impact of pregnancy on viral entry factors, but we urge caution in extrapolating these results to pathogenesis in human subjects. Illustrative of this is the questionable lack of susceptibility of rats to SARS-CoV-2 infection. Furthermore, how infection alters the expression of these factors in pregnancy remains to be studied. Third, we assayed the lung only to ensure that tissue estradiol and progesterone levels reflected the elevated systemic levels of these hormones we had reported previously [21]. Therefore, to determine correlation of estradiol and progesterone levels with viral receptor expression, dedicated studies with large sample sizes are required. Fourth, though bulk gene expression studies showed downregulation of ACE2 and TMPRSS2, it is unclear if these genes were co-localized. This could be addressed with single cell RNA sequencing studies in the future. Fifth, despite upregulated innate immune surveillance, other facets of infection, such as viral clearance and activation of adaptive immunity could be adversely impacted with pregnancy as was recently shown in pregnant transgenic hACE2-chimera female mice [54], or by sex hormones *per se* [55–57]. More importantly, we only capture the baseline state of innate immune gene expression and, therefore, are unable to comment on whether such enhanced expression results in increased ability to respond to viral challenge. Furthermore, the observed changes in gene expression were smaller-in-magnitude, therefore, their biological significance may be challenged. Sixth, we provoked only the TLR7 pathway because of the selectively enhanced expression of genes in that pathway with pregnancy, and the importance of TLR7 in combating single-stranded RNA viral infections such as SARS-CoV-2 and IAV [49–51,58–61]. Because the TLR3 pathway is more relevant for double-stranded RNA viral infections and was relatively unchanged by pregnancy, we did not perform stimulation studies with the TLR3 agonist poly I: C. Finally, we chose the third trimester because of the known susceptibility to and severity of respiratory viral infections with advanced gestation [2,62,63]. Therefore, further work is needed to determine the expression of cell entry factors across the three trimesters of pregnancy and the postpartum period to better understand the evolution of changes in viral entry mechanisms and their resolution after completion of pregnancy.

Collectively, our data points to the possibility of differences in viral entry as a potential factor in the disparate clinical presentation of pandemic influenza A and COVID-19 in pregnancy. Live virus studies in pregnant animal models along with high-resolution single cell studies of the nasal transcriptome and proteome in pregnant women are urgently needed to advance the science surrounding host-pathogen interaction in this poorly studied demographic subset.

## Supporting information

S1 File Supporting information file contains uncropped western blot files for ACE2 protein (S1 Fig), TMPRSS2 protein (S2 Fig), ST3GAL4 (S3 Fig), ST6GAL1 (S4 Fig), and immune cell frequencies in the pregnant lung (S5 Fig). (DOCX)

## References

[1] Jamieson DJ, Honein MA, Rasmussen SA, Williams JL, Swerdlow DL, Biggerstaff MS, et al. H1N1 2009 influenza virus infection during pregnancy in the USA. Lancet. 2009;374(9688):451–8. Epub 2009/08/01. doi: 10.1016/S0140-6736(09)61304-0 . 19643469

[2] Louie JK, Acosta M, Jamieson DJ, Honein MA, California Pandemic Working G. Severe 2009 H1N1 influenza in pregnant and postpartum women in California. N Engl J Med. 2010;362(1):27–35. Epub 2009/12/25. doi: 10.1056/NEJMoa0910444 . 20032319

[3] Sutton D, Fuchs K, D’Alton M, Goffman D. Universal Screening for SARS-CoV-2 in Women Admitted for Delivery. N Engl J Med. 2020. Epub 2020/04/14. doi: 10.1056/NEJMc2009316 . 32283004 PMC7175422

[4] Kelly JC, Raghuraman N, Carter EB, Palanisamy A, Stout MJ. Preprocedural asymptomatic coronavirus disease 2019 cases in obstetrical and surgical units. Am J Obstet Gynecol. 2021;224(1):114–6. Epub 20200921. doi: 10.1016/j.ajog.2020.09.023 ; PubMed Central PMCID: PMC7505049. 32971011 PMC7505049

[5] Yanes-Lane M, Winters N, Fregonese F, Bastos M, Perlman-Arrow S, Campbell JR, et al. Proportion of asymptomatic infection among COVID-19 positive persons and their transmission potential: A systematic review and meta-analysis. PLoS One. 2020;15(11):e0241536. Epub 20201103. doi: 10.1371/journal.pone.0241536 ; PubMed Central PMCID: PMC7608887. 33141862 PMC7608887

[6] Katz D, Bateman BT, Kjaer K, Turner DP, Spence NZ, Habib AS, et al. The Society for Obstetric Anesthesia and Perinatology Coronavirus Disease 2019 Registry: An Analysis of Outcomes Among Pregnant Women Delivering During the Initial Severe Acute Respiratory Syndrome Coronavirus-2 Outbreak in the United States. Anesth Analg. 2021;133(2):462–73. doi: 10.1213/ANE.0000000000005592 . 33830956

[7] Knight M, Bunch K, Vousden N, Morris E, Simpson N, Gale C, et al. Characteristics and outcomes of pregnant women admitted to hospital with confirmed SARS-CoV-2 infection in UK: national population based cohort study. BMJ. 2020;369:m2107. Epub 20200608. doi: 10.1136/bmj.m2107 ; PubMed Central PMCID: PMC7277610. 32513659 PMC7277610

[8] Overtoom EM, Rosman AN, Zwart JJ, Vogelvang TE, Schaap TP, van den Akker T, et al. SARS-CoV-2 infection in pregnancy during the first wave of COVID-19 in the Netherlands: a prospective nationwide population-based cohort study (NethOSS). BJOG. 2022;129(1):91–100. Epub 20210926. doi: 10.1111/1471-0528.16903 ; PubMed Central PMCID: PMC8652526. 34494694 PMC8652526

[9] Panagiotakopoulos L, Myers TR, Gee J, Lipkind HS, Kharbanda EO, Ryan DS, et al. SARS-CoV-2 Infection Among Hospitalized Pregnant Women: Reasons for Admission and Pregnancy Characteristics—Eight U.S. Health Care Centers, March 1-May 30, 2020. MMWR Morb Mortal Wkly Rep. 2020;69(38):1355–9. Epub 20200923. doi: 10.15585/mmwr.mm6938e2 ; PubMed Central PMCID: PMC7727498. 32970660 PMC7727498

[10] Pierce-Williams RAM, Burd J, Felder L, Khoury R, Bernstein PS, Avila K, et al. Clinical course of severe and critical coronavirus disease 2019 in hospitalized pregnancies: a United States cohort study. Am J Obstet Gynecol MFM. 2020;2(3):100134. Epub 20200508. doi: 10.1016/j.ajogmf.2020.100134 ; PubMed Central PMCID: PMC7205698. 32391519 PMC7205698

[11] Villar J, Ariff S, Gunier RB, Thiruvengadam R, Rauch S, Kholin A, et al. Maternal and Neonatal Morbidity and Mortality Among Pregnant Women With and Without COVID-19 Infection: The INTERCOVID Multinational Cohort Study. JAMA Pediatr. 2021;175(8):817–26. doi: 10.1001/jamapediatrics.2021.1050 ; PubMed Central PMCID: PMC8063132. 33885740 PMC8063132

[12] Peju E, Belicard F, Silva S, Hraiech S, Painvin B, Kamel T, et al. Management and outcomes of pregnant women admitted to intensive care unit for severe pneumonia related to SARS-CoV-2 infection: the multicenter and international COVIDPREG study. Intensive Care Med. 2022. Epub 20220817. doi: 10.1007/s00134-022-06833-8 ; PubMed Central PMCID: PMC9383668. 35978137 PMC9383668

[13] Chan KH, Zhang AJ, To KK, Chan CC, Poon VK, Guo K, et al. Wild type and mutant 2009 pandemic influenza A (H1N1) viruses cause more severe disease and higher mortality in pregnant BALB/c mice. PLoS One. 2010;5(10):e13757. Epub 20101029. doi: 10.1371/journal.pone.0013757 ; PubMed Central PMCID: PMC2966430. 21060798 PMC2966430

[14] Marcelin G, Aldridge JR, Duan S, Ghoneim HE, Rehg J, Marjuki H, et al. Fatal outcome of pandemic H1N1 2009 influenza virus infection is associated with immunopathology and impaired lung repair, not enhanced viral burden, in pregnant mice. J Virol. 2011;85(21):11208–19. Epub 20110824. doi: 10.1128/JVI.00654-11 ; PubMed Central PMCID: PMC3194964. 21865394 PMC3194964

[15] Robinson DP, Hall OJ, Nilles TL, Bream JH, Klein SL. 17beta-estradiol protects females against influenza by recruiting neutrophils and increasing virus-specific CD8 T cell responses in the lungs. J Virol. 2014;88(9):4711–20. Epub 20140212. doi: 10.1128/JVI.02081-13 ; PubMed Central PMCID: PMC3993800. 24522912 PMC3993800

[16] Vermillion MS, Nelson A, Vom Steeg L, Loube J, Mitzner W, Klein SL. Pregnancy preserves pulmonary function following influenza virus infection in C57BL/6 mice. Am J Physiol Lung Cell Mol Physiol. 2018;315(4):L517–L25. Epub 20180531. doi: 10.1152/ajplung.00066.2018 ; PubMed Central PMCID: PMC6230880. 29847990 PMC6230880

[17] Baristaite G, Gurwitz D. Estradiol reduces ACE2 and TMPRSS2 mRNA levels in A549 human lung epithelial cells. Drug Dev Res. 2022;83(4):961–6. Epub 20220201. doi: 10.1002/ddr.21923 ; PubMed Central PMCID: PMC9015589. 35103351 PMC9015589

[18] Kennedy TG, Emmens CW. Effects of estrogen and progesterone on uterine sialic acid in ovariectomized rats. Steroids. 1975;25(2):285–95. Epub 1975/02/01. doi: 10.1016/s0039-128x(75)90220-2 . 1118868

[19] Lemes RMR, Costa AJ, Bartolomeo CS, Bassani TB, Nishino MS, Pereira G, et al. 17beta-estradiol reduces SARS-CoV-2 infection in vitro. Physiol Rep. 2021;9(2):e14707. doi: 10.14814/phy2.14707 ; PubMed Central PMCID: PMC7814496. 33463909 PMC7814496

[20] Hall OJ, Limjunyawong N, Vermillion MS, Robinson DP, Wohlgemuth N, Pekosz A, et al. Progesterone-Based Therapy Protects Against Influenza by Promoting Lung Repair and Recovery in Females. PLoS Pathog. 2016;12(9):e1005840. Epub 20160915. doi: 10.1371/journal.ppat.1005840 ; PubMed Central PMCID: PMC5025002. 27631986 PMC5025002

[21] Giri T, Panda S, Kelly JC, Pancaro C, Palanisamy A. Upregulated influenza A viral entry factors and enhanced interferon-alpha response in the nasal epithelium of pregnant rats. Heliyon. 2022;8(5):e09407. Epub 20220511. doi: 10.1016/j.heliyon.2022.e09407 ; PubMed Central PMCID: PMC9111991. 35592667 PMC9111991

[22] Palanisamy A, Giri T. Reduced severe acute respiratory syndrome coronavirus 2 entry factors and enhanced innate immune gene expression in the nasal epithelium of pregnant rats. Am J Obstet Gynecol. 2021;224(1):118–20. Epub 2020/10/12. doi: 10.1016/j.ajog.2020.10.010 ; PubMed Central PMCID: PMC7544630. 33039392 PMC7544630

[23] Soldin OP, Guo T, Weiderpass E, Tractenberg RE, Hilakivi-Clarke L, Soldin SJ. Steroid hormone levels in pregnancy and 1 year postpartum using isotope dilution tandem mass spectrometry. Fertil Steril. 2005;84(3):701–10. doi: 10.1016/j.fertnstert.2005.02.045 ; PubMed Central PMCID: PMC3640374. 16169406 PMC3640374

[24] Stelzig KE, Canepa-Escaro F, Schiliro M, Berdnikovs S, Prakash YS, Chiarella SE. Estrogen regulates the expression of SARS-CoV-2 receptor ACE2 in differentiated airway epithelial cells. Am J Physiol Lung Cell Mol Physiol. 2020;318(6):L1280–L1. Epub 2020/05/21. doi: 10.1152/ajplung.00153.2020 ; PubMed Central PMCID: PMC7276982. 32432918 PMC7276982

[25] Butts CL, Shukair SA, Duncan KM, Bowers E, Horn C, Belyavskaya E, et al. Progesterone inhibits mature rat dendritic cells in a receptor-mediated fashion. Int Immunol. 2007;19(3):287–96. Epub 20070207. doi: 10.1093/intimm/dxl145 . 17289656

[26] Engels G, Hierweger AM, Hoffmann J, Thieme R, Thiele S, Bertram S, et al. Pregnancy-Related Immune Adaptation Promotes the Emergence of Highly Virulent H1N1 Influenza Virus Strains in Allogenically Pregnant Mice. Cell Host Microbe. 2017;21(3):321–33. Epub 2017/03/11. doi: 10.1016/j.chom.2017.02.020 . 28279344

[27] Robinson DP, Klein SL. Pregnancy and pregnancy-associated hormones alter immune responses and disease pathogenesis. Horm Behav. 2012;62(3):263–71. Epub 2012/03/13. doi: 10.1016/j.yhbeh.2012.02.023 ; PubMed Central PMCID: PMC3376705. 22406114 PMC3376705

[28] Kalidhindi RSR, Borkar NA, Ambhore NS, Pabelick CM, Prakash YS, Sathish V. Sex steroids skew ACE2 expression in human airway: a contributing factor to sex differences in COVID-19? Am J Physiol Lung Cell Mol Physiol. 2020;319(5):L843–L7. Epub 20200930. doi: 10.1152/ajplung.00391.2020 ; PubMed Central PMCID: PMC7789973. 32996784 PMC7789973

[29] Flerlage T, Boyd DF, Meliopoulos V, Thomas PG, Schultz-Cherry S. Influenza virus and SARS-CoV-2: pathogenesis and host responses in the respiratory tract. Nat Rev Microbiol. 2021;19(7):425–41. Epub 20210406. doi: 10.1038/s41579-021-00542-7 ; PubMed Central PMCID: PMC8023351. 33824495 PMC8023351

[30] Sungnak W, Huang N, Becavin C, Berg M, Queen R, Litvinukova M, et al. SARS-CoV-2 entry factors are highly expressed in nasal epithelial cells together with innate immune genes. Nat Med. 2020. Epub 2020/04/25. doi: 10.1038/s41591-020-0868-6 . 32327758 PMC8637938

[31] Asselin-Paturel C, Brizard G, Chemin K, Boonstra A, O’Garra A, Vicari A, et al. Type I interferon dependence of plasmacytoid dendritic cell activation and migration. J Exp Med. 2005;201(7):1157–67. Epub 20050328. doi: 10.1084/jem.20041930 ; PubMed Central PMCID: PMC2213121. 15795237 PMC2213121

[32] Jha A, Thwaites RS, Tunstall T, Kon OM, Shattock RJ, Hansel TT, et al. Increased nasal mucosal interferon and CCL13 response to a TLR7/8 agonist in asthma and allergic rhinitis. J Allergy Clin Immunol. 2021;147(2):694–703 e12. Epub 20200724. doi: 10.1016/j.jaci.2020.07.012 . 32717253

[33] Xirakia C, Koltsida O, Stavropoulos A, Thanassopoulou A, Aidinis V, Sideras P, et al. Toll-like receptor 7-triggered immune response in the lung mediates acute and long-lasting suppression of experimental asthma. Am J Respir Crit Care Med. 2010;181(11):1207–16. Epub 20100311. doi: 10.1164/rccm.200908-1255OC . 20224068

[34] Wang H, Leigh J. Effects of nitric oxide synthase inhibitor omega-nitro-L-arginine methyl ester, on silica-induced inflammatory reaction and apoptosis. Part Fibre Toxicol. 2006;3:14. Epub 20061107. doi: 10.1186/1743-8977-3-14 ; PubMed Central PMCID: PMC1636655. 17090306 PMC1636655

[35] Loske J, Rohmel J, Lukassen S, Stricker S, Magalhaes VG, Liebig J, et al. Pre-activated antiviral innate immunity in the upper airways controls early SARS-CoV-2 infection in children. Nat Biotechnol. 2022;40(3):319–24. Epub 20210818. doi: 10.1038/s41587-021-01037-9 . 34408314

[36] Crook M, Constable S, Lumb P, Rymer J. Elevated serum sialic acid in pregnancy. J Clin Pathol. 1997;50(6):494–5. Epub 1997/06/01. doi: 10.1136/jcp.50.6.494 ; PubMed Central PMCID: PMC499983. 9378816 PMC499983

[37] Jansen BC, Bondt A, Reiding KR, Lonardi E, de Jong CJ, Falck D, et al. Pregnancy-associated serum N-glycome changes studied by high-throughput MALDI-TOF-MS. Sci Rep. 2016;6:23296. Epub 2016/04/15. doi: 10.1038/srep23296 ; PubMed Central PMCID: PMC4831011. 27075729 PMC4831011

[38] Rajan R, Sheth AR, Rao SS. Sialic acid, sialyltransferase and neuraminidase levels in maternal plasma, urine and lymphocytes during pregnancy and post-partum period—a longitudinal study in women. Eur J Obstet Gynecol Reprod Biol. 1983;16(1):37–46. Epub 1983/09/01. doi: 10.1016/0028-2243(83)90218-6 . 6628818

[39] de Graaf M, Fouchier RA. Role of receptor binding specificity in influenza A virus transmission and pathogenesis. EMBO J. 2014;33(8):823–41. Epub 20140325. doi: 10.1002/embj.201387442 ; PubMed Central PMCID: PMC4194109. 24668228 PMC4194109

[40] Imai M, Kawaoka Y. The role of receptor binding specificity in interspecies transmission of influenza viruses. Curr Opin Virol. 2012;2(2):160–7. Epub 20120324. doi: 10.1016/j.coviro.2012.03.003 ; PubMed Central PMCID: PMC5605752. 22445963 PMC5605752

[41] Thompson AJ, Paulson JC. Adaptation of influenza viruses to human airway receptors. J Biol Chem. 2021;296:100017. Epub 20201122. doi: 10.1074/jbc.REV120.013309 ; PubMed Central PMCID: PMC7948470. 33144323 PMC7948470

[42] Boon AC, Finkelstein D, Zheng M, Liao G, Allard J, Klumpp K, et al. H5N1 influenza virus pathogenesis in genetically diverse mice is mediated at the level of viral load. mBio. 2011;2(5). Epub 20110906. doi: 10.1128/mBio.00171-11 ; PubMed Central PMCID: PMC3171982. 21896679 PMC3171982

[43] de Jong MD, Simmons CP, Thanh TT, Hien VM, Smith GJ, Chau TN, et al. Fatal outcome of human influenza A (H5N1) is associated with high viral load and hypercytokinemia. Nat Med. 2006;12(10):1203–7. Epub 20060910. doi: 10.1038/nm1477 ; PubMed Central PMCID: PMC4333202. 16964257 PMC4333202

[44] Fajnzylber J, Regan J, Coxen K, Corry H, Wong C, Rosenthal A, et al. SARS-CoV-2 viral load is associated with increased disease severity and mortality. Nat Commun. 2020;11(1):5493. Epub 20201030. doi: 10.1038/s41467-020-19057-5 ; PubMed Central PMCID: PMC7603483. 33127906 PMC7603483

[45] Hijano DR, Brazelton de Cardenas J, Maron G, Garner CD, Ferrolino JA, Dallas RH, et al. Clinical correlation of influenza and respiratory syncytial virus load measured by digital PCR. PLoS One. 2019;14(9):e0220908. Epub 20190903. doi: 10.1371/journal.pone.0220908 ; PubMed Central PMCID: PMC6720028. 31479459 PMC6720028

[46] Lee N, Chan PK, Hui DS, Rainer TH, Wong E, Choi KW, et al. Viral loads and duration of viral shedding in adult patients hospitalized with influenza. J Infect Dis. 2009;200(4):492–500. doi: 10.1086/600383 ; PubMed Central PMCID: PMC7110250. 19591575 PMC7110250

[47] Magleby R, Westblade LF, Trzebucki A, Simon MS, Rajan M, Park J, et al. Impact of Severe Acute Respiratory Syndrome Coronavirus 2 Viral Load on Risk of Intubation and Mortality Among Hospitalized Patients With Coronavirus Disease 2019. Clin Infect Dis. 2021;73(11):e4197–e205. doi: 10.1093/cid/ciaa851 ; PubMed Central PMCID: PMC7337625. 32603425 PMC7337625

[48] Souverein D, van Stralen K, van Lelyveld S, van Gemeren C, Haverkort M, Snijders D, et al. Initial Severe Acute Respiratory Syndrome Coronavirus 2 Viral Load Is Associated With Disease Severity: A Retrospective Cohort Study. Open Forum Infect Dis. 2022;9(7):ofac223. Epub 20220501. doi: 10.1093/ofid/ofac223 ; PubMed Central PMCID: PMC9272435. 35821732 PMC9272435

[49] Asano T, Boisson B, Onodi F, Matuozzo D, Moncada-Velez M, Maglorius Renkilaraj MRL, et al. X-linked recessive TLR7 deficiency in ~1% of men under 60 years old with life-threatening COVID-19. Sci Immunol. 2021;6(62). doi: 10.1126/sciimmunol.abl4348 ; PubMed Central PMCID: PMC8532080. 34413140 PMC8532080

[50] Fallerini C, Daga S, Mantovani S, Benetti E, Picchiotti N, Francisci D, et al. Association of Toll-like receptor 7 variants with life-threatening COVID-19 disease in males: findings from a nested case-control study. Elife. 2021;10. Epub 20210302. doi: 10.7554/eLife.67569 ; PubMed Central PMCID: PMC7987337. 33650967 PMC7987337

[51] Mantovani S, Daga S, Fallerini C, Baldassarri M, Benetti E, Picchiotti N, et al. Rare variants in Toll-like receptor 7 results in functional impairment and downregulation of cytokine-mediated signaling in COVID-19 patients. Genes Immun. 2022;23(1):51–6. Epub 20211224. doi: 10.1038/s41435-021-00157-1 ; PubMed Central PMCID: PMC8703210. 34952932 PMC8703210

[52] Karupiah G, Chen JH, Mahalingam S, Nathan CF, MacMicking JD. Rapid interferon gamma-dependent clearance of influenza A virus and protection from consolidating pneumonitis in nitric oxide synthase 2-deficient mice. J Exp Med. 1998;188(8):1541–6. doi: 10.1084/jem.188.8.1541 ; PubMed Central PMCID: PMC2213404. 9782132 PMC2213404

[53] Perrone LA, Belser JA, Wadford DA, Katz JM, Tumpey TM. Inducible nitric oxide contributes to viral pathogenesis following highly pathogenic influenza virus infection in mice. J Infect Dis. 2013;207(10):1576–84. Epub 20130218. doi: 10.1093/infdis/jit062 . 23420903

[54] Zhu G, Du S, Wang Y, Huang X, Hu G, Lu X, et al. Delayed Antiviral Immune Responses in Severe Acute Respiratory Syndrome Coronavirus Infected Pregnant Mice. Front Microbiol. 2021;12:806902. Epub 20220121. doi: 10.3389/fmicb.2021.806902 ; PubMed Central PMCID: PMC8814454. 35126335 PMC8814454

[55] Kadel S, Kovats S. Sex Hormones Regulate Innate Immune Cells and Promote Sex Differences in Respiratory Virus Infection. Front Immunol. 2018;9:1653. Epub 20180720. doi: 10.3389/fimmu.2018.01653 ; PubMed Central PMCID: PMC6062604. 30079065 PMC6062604

[56] Klein SL, Flanagan KL. Sex differences in immune responses. Nat Rev Immunol. 2016;16(10):626–38. Epub 20160822. doi: 10.1038/nri.2016.90 . 27546235

[57] Scully EP, Haverfield J, Ursin RL, Tannenbaum C, Klein SL. Considering how biological sex impacts immune responses and COVID-19 outcomes. Nat Rev Immunol. 2020;20(7):442–7. Epub 20200611. doi: 10.1038/s41577-020-0348-8 ; PubMed Central PMCID: PMC7288618. 32528136 PMC7288618

[58] Liong S, Oseghale O, To EE, Brassington K, Erlich JR, Luong R, et al. Influenza A virus causes maternal and fetal pathology via innate and adaptive vascular inflammation in mice. Proc Natl Acad Sci U S A. 2020;117(40):24964–73. Epub 2020/09/23. doi: 10.1073/pnas.2006905117 ; PubMed Central PMCID: PMC7547222. 32958663 PMC7547222

[59] van der Made CI, Simons A, Schuurs-Hoeijmakers J, van den Heuvel G, Mantere T, Kersten S, et al. Presence of Genetic Variants Among Young Men With Severe COVID-19. JAMA. 2020;324(7):663–73. doi: 10.1001/jama.2020.13719 ; PubMed Central PMCID: PMC7382021. 32706371 PMC7382021

[60] Makris S, Johansson C. R848 or influenza virus can induce potent innate immune responses in the lungs of neonatal mice. Mucosal Immunol. 2021;14(1):267–76. Epub 20200623. doi: 10.1038/s41385-020-0314-6 ; PubMed Central PMCID: PMC7116567. 32576926 PMC7116567

[61] Zhang Q, Matuozzo D, Le Pen J, Lee D, Moens L, Asano T, et al. Recessive inborn errors of type I IFN immunity in children with COVID-19 pneumonia. J Exp Med. 2022;219(8). Epub 20220616. doi: 10.1084/jem.20220131 ; PubMed Central PMCID: PMC9206114. 35708626 PMC9206114

[62] Holstein R, Dawood FS, O’Halloran A, Cummings C, Ujamaa D, Daily Kirley P, et al. Characteristics and Outcomes of Hospitalized Pregnant Women With Influenza, 2010 to 2019: A Repeated Cross-Sectional Study. Ann Intern Med. 2022;175(2):149–58. Epub 20211228. doi: 10.7326/M21-3668 . 34958603

[63] Schell RC, Macias DA, Garner MWH, White AM, McIntire DD, Pruszynski J, et al. Examining the impact of trimester of diagnosis on COVID-19 disease progression in pregnancy. Am J Obstet Gynecol MFM. 2022:100728. Epub 20220819. doi: 10.1016/j.ajogmf.2022.100728 ; PubMed Central PMCID: PMC9391234. 35995369 PMC9391234

