# Supplementary material for: Pregnancy-induced differential expression of SARS-CoV-2 and influenza a viral entry factors in the lower respiratory tract
Source: PLoS One. 2023 Jul 12;18(7):e0281033. doi: 10.1371/journal.pone.0281033 (PMC10337962; doi:10.1371/journal.pone.0281033)
Supplement: S1 File — (DOCX) [file pone.0281033.s001.docx]

**SUPPORTING INFORMATION FILE**

**Pregnancy-induced differential expression of SARS-CoV-2 and influenza A viral entry factors in the lower respiratory tract**

Tusar Giri^1^, Santosh Panda^2^, Arvind Palanisamy^1,3*^

^1^Department of Anesthesiology, Washington University School of Medicine, St. Louis, MO

^2^Department of Pathology, Washington University School of Medicine, St. Louis, MO

^3^Department of Obstetrics and Gynecology, Washington University School of Medicine, St. Louis, MO

**^*^Corresponding author**

Arvind Palanisamy, MD, FRCA

Department of Anesthesiology

Department of Obstetrics and Gynecology

Washington University School of Medicine

660 S Euclid Ave

St. Louis, MO, 63110

Telephone: 314-362-2628

Email: [arvind.palanisamy@wustl.edu](mailto:arvind.palanisamy@wustl.edu)

**S1 Fig. Uncropped western blot files for ACE2 protein**

rat intestine

hACE2

MW

Pregnant

Non-pregnant

**
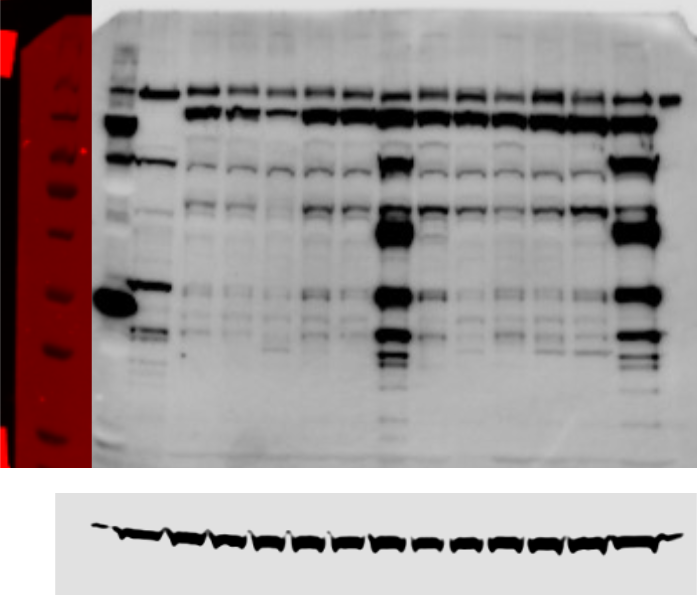
**

β-actin

ACE2 (119 kD)

119 kD

199 kD

84 kD

57 kD

39 kD

50 kD

28 kD

22 kD

15 kD

**S1 Fig. Uncropped western blot files for ACE2 protein.** Please note that the order of samples is reversed in the full-length blot (pregnant followed by non-pregnant samples). Molecular weights indicated on the left. Lane 1: molecular weight marker, Lane 2: rat intestinal lysate as positive control, Lanes 3-8: pregnant lung homogenates, Lanes 9-14: non-pregnant lung homogenates, Lane 15: hACE2 positive control. β-actin (below) was used as loading control.

**S2 Fig. Uncropped western blot files for TMPRSS2 protein**

+ control TMPRSS2

rat intestine

MW

Non-pregnant

Pregnant

**
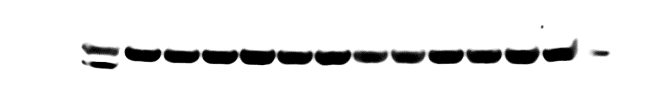

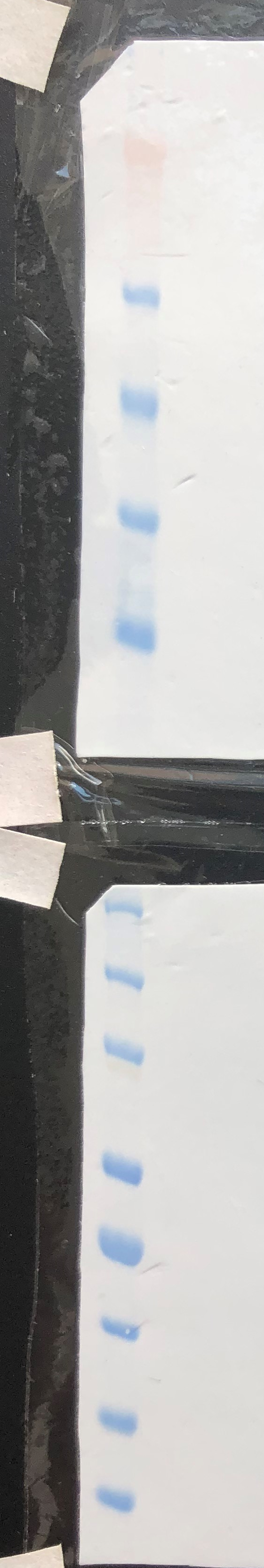

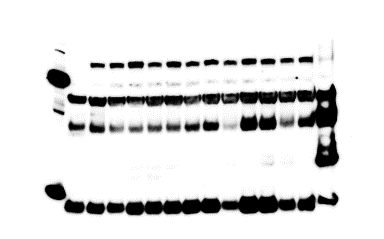
**

84 kD

57 kD

22 kD

119 kD

28 kD

199 kD

50 kD

39 kD

TMPRSS2

(70 kD)

β-actin

**Densitometric quantification**

**
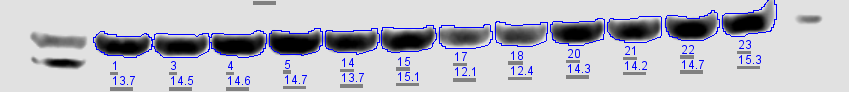
**

**
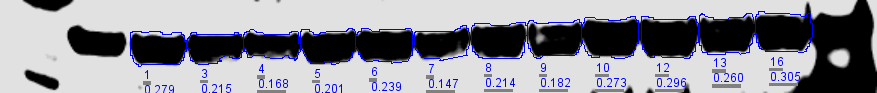
**

TMPRSS2

(70 kD)

β-actin

**S2 Fig. Uncropped western blot files for TMPRSS2 protein.** Please note that the order of samples is reversed in the full-length blot (pregnant followed by non-pregnant samples). Molecular weights indicated on the right. Lane 1: molecular weight marker, Lane 2: rat intestinal lysate as positive control, Lanes 3-8: pregnant lung homogenates, Lanes 9-14: non-pregnant lung homogenates, Lane 15: TMPRSS2 expressed in HEK293 cells as positive control. β-actin (below) was used as loading control. Densitometric quantification values for TMPRSS2 and β-actin provided below the immunoblots.

**S3 Fig. Uncropped western blot files for ST3GAL4**

+ control

(rat intestine)

ST3GAL4 (38 kD)

Pregnant

Non-pregnant

11 kD

15 kD

50 kD

40 kD

60 kD

85 kD

120 kD

MW

**
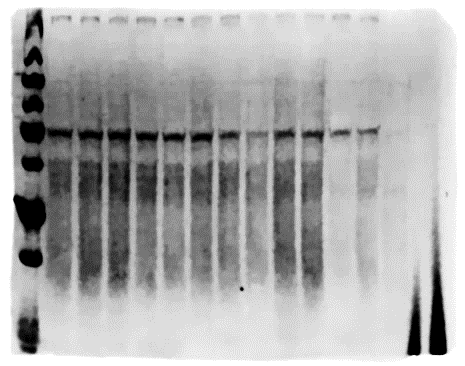
**

24 kD

28 kD

**
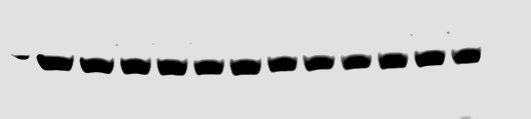
**

β-actin

**S3 Fig. Uncropped western blot files for ST3GAL4 protein.** Please note that the order of samples is reversed in the full-length blot (pregnant followed by non-pregnant samples). Molecular weights indicated on the left. Lane 1: molecular weight marker, Lanes 2-7: pregnant lung homogenates, Lanes 8-13: non-pregnant lung homogenates, Lanes 14-15: technical difficulties with gel electrophoresis for rat intestinal lysate as positive control. β-actin (below) was used as loading control. Please note missing β-actin bands in lanes 14-15 indicating technical problems.

**S4 Fig. Uncropped western blot files for ST6GAL1**

rat intestine

MW

Non-pregnant

Pregnant


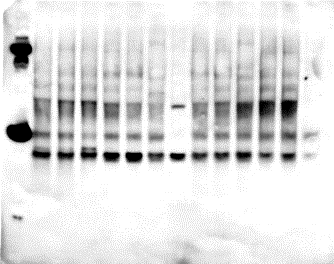


ST6GAL1 (21 kD)


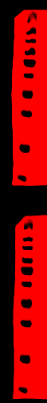

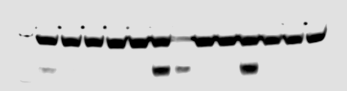


199 kD

119 kD

84 kD

57 kD

50 kD

39 kD

28 kD

22 kD

15 kD

10 kD

β-actin

**S4 Fig. Uncropped western blot files for ST6GAL1 protein.** Please note that the order of samples is reversed in the full-length blot (pregnant followed by non-pregnant samples). Molecular weights indicated on the left. Lane 1: molecular weight marker, Lanes 2-7: pregnant lung homogenates, Lane 8: rat intestinal lysate as positive control, Lanes 9-14: non-pregnant lung homogenates. β-actin (below) was used as loading control.

**S5 Fig. Immune cell frequencies in the pregnant lung**

**
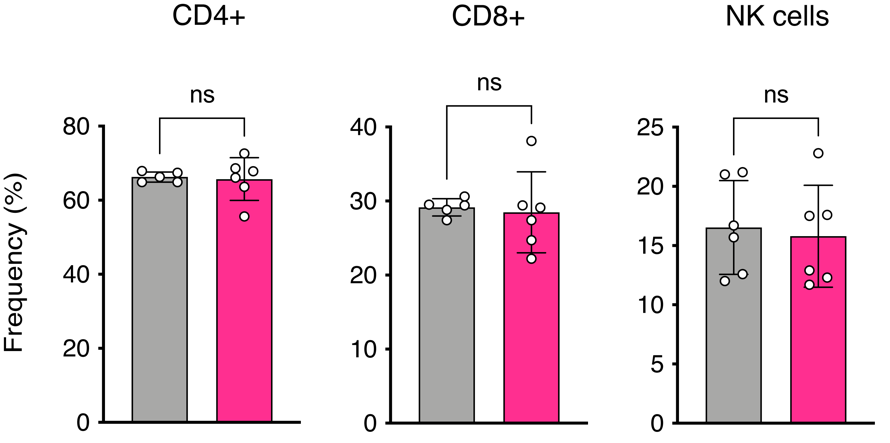
**

**S5 Fig. Immune cell frequencies in the pregnant lung.** No differences were observed in the frequencies of CD4+, CD8+, and NK cells between non-pregnant (gray bar) and pregnant (pink bar) samples. Data were analyzed with Welch’s t-test and presented as mean ± SD (n=6 per condition).
